# Outcomes and safety of antibiotic-loaded calcium sulfate beads in orthopaedics: A systematic review and meta-analysis

**DOI:** 10.1016/j.jor.2026.04.019

**Published:** 2026-04-28

**Authors:** Alberto Pedrazzini, Vishwa Suravaram, Victor Yan Zhe Lu, Armando Hoch, Patrick O. Zingg, Octavian Andronic

**Affiliations:** a Department of Orthopaedics, Balgrist University Hospital, University of Zurich, Zurich, Switzerland; b Department of Orthopaedics, Fiona Stanley Hospital, Perth, WA, Australia; c Department of Orthopaedics and Traumatology, Chinese University of Hong Kong, Sha Tin, Hong Kong, China

**Keywords:** Calcium sulfate, Antibiotic beads, Infection, Osteomyelitis, Periprosthetic joint infection, Prevention

## Abstract

**Introduction:**

Calcium sulfate (CS) has emerged as a local antibiotic delivery system, offering biodegradability, osteoconductive properties and sustained antibiotic release. The aim was to evaluate the clinical outcomes and safety of antibiotic-loaded CS beads as an adjunct in the management and prevention of orthopaedic infections.

**Methods:**

The systematic review followed the Preferred Reporting Items for Systematic Reviews and Meta-Analyses guidelines. Studies reporting outcomes on the use of antibiotic-loaded CS beads in orthopaedics were considered eligible. Risk of bias assessment was performed using the methodological index for non-randomized studies (MINORS) criteria. Evaluated outcomes included rates of infection eradication or incidence, bone union, CS resorption, complications and reinterventions. Exploratory meta-analyses used a random-effects model, with heterogeneity assessed via the I^2^ statistic.

**Results:**

The systematic search included 41 studies. No randomized controlled trials were identified. Twenty-three studies examined patients with osteomyelitis, yielding a pooled infection eradication rate of 88% (95% CI: 85 to 90%; I^2^ = 19%). Among patients with infected nonunion, the pooled bone union rate was 93% (95% CI: 85 to 97%; I^2^ = 36%). In patients with periprosthetic joint infections (PJI), the pooled infection eradication rate reached 88% (95% CI: 76 to 95%; I^2^ = 87%). Wound-related complications were documented in 17% (189/1086) of osteomyelitis cases, compared to 8% (23/277) of PJI cases. PJI treatment was associated with minimal complications, including 12 cases of hypercalcemia (12/277, 4%) and nine cases of heterotopic ossification (9/262, 3%).

**Conclusion:**

Current evidence describes favorable infection eradication rates when antibiotic-loaded CS beads are used as an adjunct in orthopaedic infection management and prevention, but the absence of comparative trials precludes conclusions regarding independent efficacy. All studies showed complete CS resorption. Wound-related complications were substantial in osteomyelitis cases, while other adverse events were rare. Higher-quality comparative studies are required before routine adoption.

PROSPERO registration number: CRD42023489317.

## 1 Introduction

Infections in orthopaedic and trauma surgery, ranging from superficial soft tissue infections to osteomyelitis and periprosthetic joint infections (PJI), constitute major causes of morbidity and healthcare burden.^1^^,^^2^ Recent epidemiological data revealed a growing trend in osteomyelitis prevalence.^3^ At the same time, the increasing volume of arthroplasty procedures, particularly total hip arthroplasty (THA) and total knee arthroplasty (TKA), has contributed to a corresponding rise of PJI.^4^ The emergence of resistant bacteria further complicates treatment,^5^ underscoring the need for innovative treatment strategies.

The management of osteomyelitis has historically relied on surgical debridement and antibiotic therapy.^6^ PJI treatment includes complete prosthesis revision, either as one- or two-staged procedure, or debridement with exchange of modular components accompanied by appropriate antibiotic therapy, usually referred to as DAIR (Debridement, Antibiotics and Implant Retention). Despite technical advances, considerable failure rates have been reported.^7^^,^^8^ Systemic antibiotics face limitations due to poor vascularity in affected areas, biofilm formation, difficulty achieving adequate local concentrations and potential systemic side effects.^9^

Local antibiotic delivery systems have emerged as an important treatment option. Polymethyl methacrylate (PMMA) cement is commonly used but presents limitations, including rapid antibiotic elution, after which it may act as a nidus for bacterial proliferation and require surgical removal.^10^^,^^11^ These drawbacks have directed attention towards biodegradable materials for local antibiotic delivery. Calcium sulfate (CS) is fully biodegradable and possesses osteoconductive properties, eliminating the need for surgical removal while simultaneously enhancing dead space management by providing a scaffold for bone regeneration.^12^ Additionally, CS can be combined with all water-soluble antibiotics and demonstrated superior elution characteristics compared to PMMA, maintaining effective concentrations over up to six weeks.^10^^,^^13^^,^^14^

Despite these favorable characteristics, concerns exist regarding prolonged wound discharge, heterotopic ossification (HO), symptomatic hypercalcemia, acute renal failure, and potential implant damage 15, 16, 17, 18.

Previous systematic reviews focused on single indications and incorporated relatively few studies ^11^^,^^19^, ^20^, ^21^, ^22^, ^23^, ^24^. Moreover, no previous literature review has addressed infection prevention. The aim of this systematic review was to perform an updated evaluation of the evidence regarding the clinical outcomes and safety profile of CS in orthopaedics.

## 2 Methods

### 2.1 Strategy of the systematic search

The systematic review followed The Preferred Reporting Items for Systematic Reviews and Meta-analysis (PRISMA) guidelines^25^ and was registered in the International Prospective Register for Systematic Reviews and Meta-analyses (PROSPERO) under the registration number: CRD42023489317. Ethics approval was waived as this study analyzed only previously published data. The systematic search strategy for peer-reviewed original articles evaluating the effectiveness of antibiotic-loaded CS beads in orthopaedic surgery covered multiple databases including MEDLINE, Web of Science, Scopus, and ProQuest. All studies published from the inception of the databases to December 31st, 2024 were included. The following keywords were used combined using the Boolean terms AND and OR: "calcium sulfate beads" OR "local antibiotics" AND "prevention" OR "prophylaxis" OR "treatment" OR "therapy" AND "prosthetic joint infections" OR "bone infections" OR "osteomyelitis" OR "soft tissue infections".

### 2.2 Selection process and data extraction

After duplicates removal, screening of studies based on titles and abstracts was performed by two authors in a blinded and independent process. Discrepancies were resolved through consensus or consultation with the senior author. Selected studies were assessed for eligibility by evaluating full texts according to the predefined inclusion and exclusion criteria (Table 1). This systematic review focused exclusively on pure CS products, such as OsteoSet® and Stimulan®, while excluding composite formulations of CS with other materials (e.g. Cerament ®, Herafill ®, PerOssal ®), thereby reducing heterogeneity of data. Extracted patient characteristics included sample size, gender distribution, mean age, anatomical site involved, and follow-up duration. Documentation also covered etiology or treatment indication, Cierny-Mader classification,^26^ identified pathogens, and details regarding CS beads (commercial product, antibiotic selection, and placement). Additionally, data on concurrent systemic antibiotic therapy and surgical procedures, clinical outcomes, complication and reintervention rates were collected.

**Table 1:** – Inclusion and exclusion criteria for study screening.

Inclusion criteria	Exclusion criteria
Antibiotic-loaded calcium sulfate beads for prevention and treatment of orthopaedic infection	Non-English articles
Publication between from database inception to December 31st, 2024	Reviews, hypothesis articles, surgical techniques, cadaveric or animal studies, case reports
Oxford Centre of Evidence-Based Medicine 2011 Levels of Evidence: I–IV	Abstract or full text not accessible
	Insufficient or inadequate data for extraction
	Diabetic foot infection related osteomyelitis
	Studies focusing on paediatric patient population
	Use of carrier materials other than pure calcium sulfate
	Use of calcium sulfate in spine surgery

Primary outcomes were infection eradication rate for studies evaluating osteomyelitis treatment, bone union rate for infected nonunions, and infection eradication rate for studies evaluating PJI treatment. Secondary outcomes included CS resorption, time to resorption, complications and reinterventions.

### 2.3 Risk of bias assessment

The methodological index for non-randomized studies (MINORS) criteria were used to assess risk of bias.^27^ MINORS evaluates eight items for noncomparative studies and an additional four for comparative studies, with each item scored 0 (not reported), 1 (reported but inadequate), or 2 (reported and adequate), yielding maximum scores of 16 and 24, respectively.

### 2.4 Statistical analysis

Meta-analysis was carried out using R version 4.2.1, for three primary outcome measures: infection eradication rate for osteomyelitis, bone union rate for infected nonunions, and infection eradication rate for PJI. Secondary outcomes were reported descriptively due to heterogeneous definitions and reporting. Since heterogeneity within the dataset was predicted, a random effects model was used. Effect sizes were pooled using the inverse-variance weighting method. The Paule-Mandel model was used to calculate the variance of distribution of effect sizes (τ^2^).^28^ Higgins and Thompson's I^2^ statistic was used to illustrate heterogeneity. Heterogeneity values above 40% were considered moderate, and those above 75% were considered highly heterogeneous.^29^ Prediction intervals were included to suggest a range into which other studies' effect size may fall.^30^^,^^31^ A *P*-value of <0.05 was used to determine statistical significance.

## 3 Results

### 3.1 Database search

**Fig. 1:**
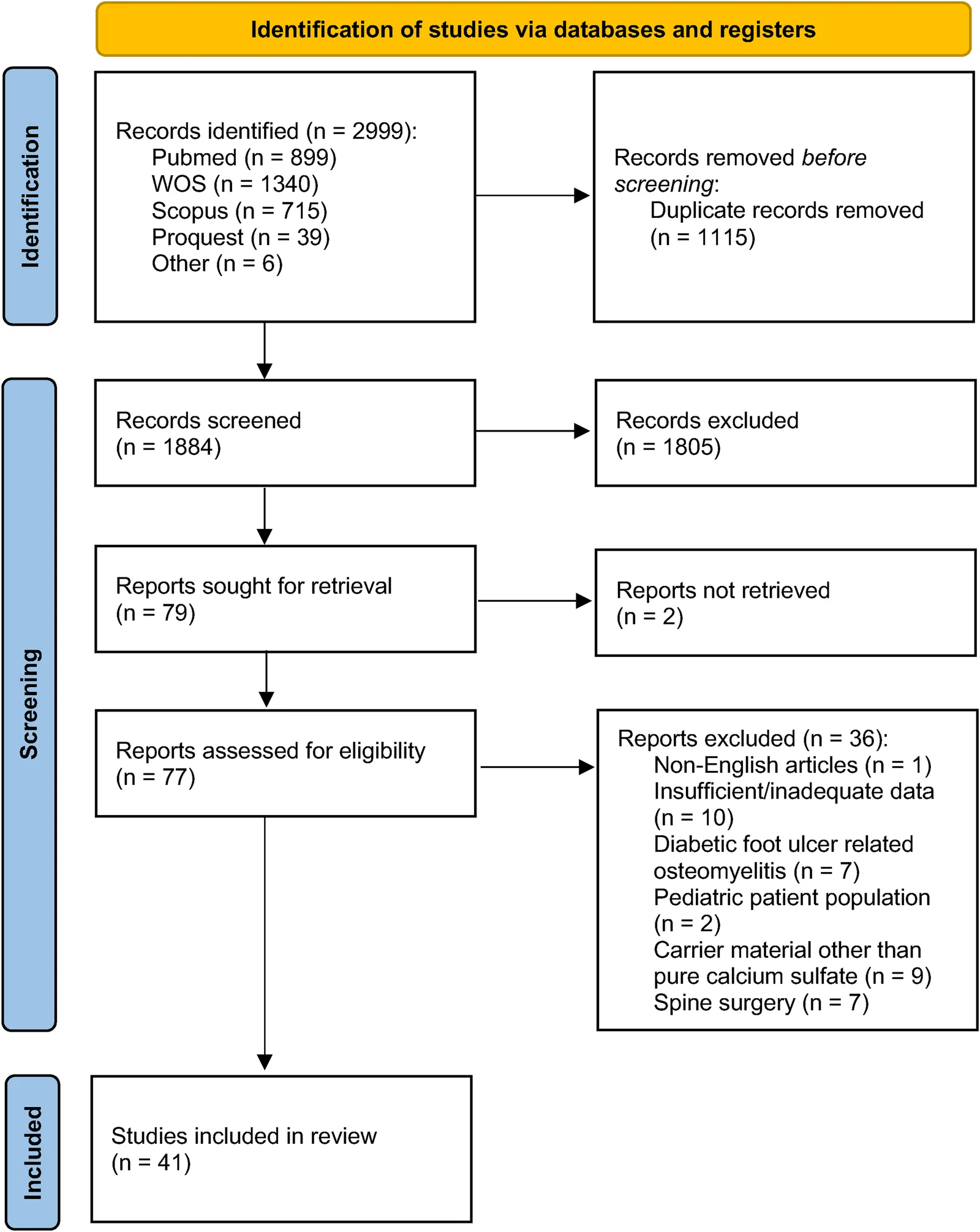
Flowchart of the systematic search.

The initial database search identified 2999 records. After duplicate removal and screening, 77 studies underwent full-text review, of which 41 were ultimately included (Fig. 1). ^15^^,^^32^, ^33^, ^34^, ^35^, ^36^, ^37^, ^38^, ^39^, ^40^, ^41^, ^42^, ^43^, ^44^, ^45^, ^46^, ^47^, ^48^, ^49^, ^50^, ^51^, ^52^, ^53^, ^54^, ^55^, ^56^, ^57^, ^58^, ^59^, ^60^, ^61^, ^62^, ^63^, ^64^, ^65^, ^66^, ^67^, ^68^, ^69^, ^70^, ^71^. Studies comprised two prospective cohort studies^35^^,^^66^ (level II), 13 retrospective cohort studies ^34^^,^^41^^,^^50^^,^^52^, ^53^, ^54^, ^55^^,^^59^^,^^60^^,^^65^^,^^68^^,^^69^^,^^71^ and one historically controlled prospective study^57^ (level III), as well as 20 retrospective case series ^33^^,^^36^, ^37^, ^38^, ^39^, ^40^^,^^42^, ^43^, ^44^^,^^47^, ^48^, ^49^^,^^51^^,^^56^^,^^58^^,^^61^^,^^62^^,^^64^^,^^67^^,^^70^ and five prospective case series^15^^,^^32^^,^^45^^,^^46^^,^^63^ (level IV). Notably, no randomized controlled trials were identified. Studies were categorized according to the indication for CS application: 23 studies examined CS in osteomyelitis treatment 32, 33, 34, 35^,^^37^^,^^38^^,^^41^^,^^43^^,^^45^^,^^47^, ^48^, ^49^, ^50^, ^51^^,^^55^^,^^56^^,^^59^^,^^61^, ^62^, ^63^, ^64^^,^^66^^,^^67^, 11 investigated PJI management,^39^^,^^40^^,^^46^^,^^52^^,^^53^^,^^57^^,^^58^^,^^60^^,^^65^^,^^69^^,^^71^ while two studies focused on PJI prophylaxis.^54^^,^^68^ Five studies reported combined cohorts encompassing more than one clinical indication for CS use without separate reporting, precluding allocation to a single category. These were classified as mixed indications.^15^^,^^36^^,^^42^^,^^44^^,^^70^

MINORS score ranged from 31 to 75%, with consistent limitations including lack of prospective sample size calculation and incomplete follow-up reporting.

### 3.2 Demographics and infection profile

#### 3.2.1 Osteomyelitis treatment

Studies on osteomyelitis treatment included 1205 patients (1208 extremities) with a weighted mean follow-up of 32.9 months (range, 6 to 85.2). The tibia was most frequently affected (531/1,078, 49%) and *Staphylococcus aureus* was the most common pathogen (311/932, 33%). Demographics and treatment characteristics are detailed in Table 2, Table 3.

**Table 2:** – Demographics of the included studies.

First Author	Year	Level of Evidence	Number of patients (extremities)	Sex	Mean age, years (range)	Bone/joint involved (n)	Mean follow-up, months (range)	MINORS score (%)
**Osteomyelitis treatment**								
McKee	2002	II	25	15 M, 10 W	43 (27 to 69)	Tibia (15), Femur (6), Humerus (1), Radius/ulna (3)	28 (20 to 38)	75
Gitelis	2002	IV	6	3 M, 3 W	50 (26 to 85)	Tibia (3), Femur (3)	28 (18 to 40)	38
Chang	2007	III	25	NA	NA	NA	>36	46
McKee	2010	II	15	10 M, 5 W	44.1 (16 to 86)	Tibia (8), Femur (4), Humerus (2), Radius/ulna (1)	>24	75
Humm	2014	IV	21	18 M, 3 W	49 (26 to 88)	Tibia (21)	16 (6 to 25)	32
Ferguson	2014	III	193 (195)	150 M, 43 W	46.1 (16.1 to 82.0)	Tibia (88), Femur (73), Humerus (10), Radius/ulna (6), Pelvis (4), Foot/ankle (10), Knee (4)	44.4 (15.6 to 85.2)	75
Ferrando	2017	IV	13	9 M, 4 W	48 (17 to 67)	Tibia (6), Femur (2), Humerus (1), Foot/ankle (4)	22 (16 to 29)	63
Masrouha	2018	IV	13	13 M	35 (18 to 63)	Tibia (9), Femur (2), Humerus (2)	24	44
Badie	2019	IV	30	25 M, 5 W	26.2 (17 to 53)	Tibia (14), Femur (11), Humerus (2), Radius/ulna (3)	>12	44
Qin	2020	IV	33	27 M, 6 W	44.5 (17 to 67)	Foot/ankle (33)	35.9 (12 to 75)	44
Zhou	2020	IV	42 (43)	24 M, 19 W	43.7 (23 to 74)	Tibia (43)	42.8 (12.8 to 77.5)	44
Jiang	2020	IV	34	27 M, 7 W	41 (3 to 67)	Foot/ankle (34)	26 (12 to 68)	44
Zhao	2020	III	10	10 M	48.2 (SD 19.2)	Tibia (5), Femur (5)	20 (SD 5.4)	63
Ruan	2021	IV	35	25 M, 10 W	54 (34 to 82)	Tibia (35)	38 (24 to 60)	44
Xu	2022	III	41	30 M, 11 W	47.3 (SD 16.2)	Foot/ankle (41)	>22	63
Patel	2023	IV	13	10 M, 3 W	38 (22 to 66)	Tibia (10), Femur (3)	19.7 (12 to 28)	56
McKee	2023	III	106	68 M, 38 W	44.2 (SD 14.2)	NA	NA	46
Du	2023	IV	145	128 M, 17 W	49.6 (20 to 84)	Tibia (78), Femur (41), Foot/ankle (15), Knee (5), Elbow (6)	NA	31
Casiraghi	2023	IV	7	4 M, 3 W	38.7 (16 to 53)	Pelvis (7)	9 (6 to 16)	38
Mereddy	2023	II	106	67 M, 33 W	50 (11 to 79)	Tibia (28), Femur (46), Humerus (4), Radius/ulna (14) Foot/ankle (6), Hip (5), Knee (2), Shoulder (2)∗	20 (12 to 30)	63
Selvaratnam	2023	IV	34	24 M, 10 W	46.8 (20 to 67)	Tibia (16), Femur (14), Humerus (3), Radius/ulna (1)	30 (16.8 to 79.2)	50
Palo	2024	II	95	59 M, 36 W	27.1 (SD 5.8)	Tibia (61), Femur (15), Humerus (19)	>12	75
Su	2024	IV	163	136 M, 27 W	51 (18 to 85)	Tibia (91), Femur (39), Humerus (6), Radius/ulna (2) Foot/ankle (15), Knee (10)	>12	56
**Summary**			1205 (1208)	882 M (75%), 298 W (25%)	45.1∗∗ (3 to 88)	Tibia (531, 49%), Femur (264, 24%), Humerus (50, 5%), Radius/ulna (30, 3%), Foot/ankle (158, 15%), Pelvis (11, 1%), Hip (2, 0.2%), Knee (24, 2%), Shoulder (2, 0.2%), Elbow (6, 0.6%)∗	32.9∗∗ (6 to 85.2)	31 to 75
Summary data excludes studies with no reported data. ∗Inconsistent report, ∗∗weighted mean (excluding studies with no reported data)								

**PJI treatment**								
Kallala	2015	IV	15	8 M, 7 W	64.8 (41 to 83)	THA (7), Hip resurfacing (2) TKA (6)	16 (12 to 22)	50
Flierl	2017	IV	32 (33)	22 M, 11 W	62 (32 to 88)	THA (6), TKA (27)	13 (3 to 30)	31
Sandiford	2020	IV	29	13 M, 16 W	67 (36 to 94)	THA (29)	1.5	56
Tarity	2022	III	20	12 M, 8 W	72.1 (SD 11.8)	THA (8), TKA (12)	>24	58
Piovan	2022	III	17	10 M, 7 W	64.2 (SD 8.7)	TKA (17)	16.1 (12 to 37)	63
Reinisch	2022	III	27	16 M, 11 W	71.3 (34.6 to 91.9)	THA (27)	>12	63
Iorio	2023	III	20	12 M, 8 W	76 (SD 10.75)	TKA (20)	>12	54
Indelli	2023	IV	62	57 M, 5 W	71 (62 to 77)	THA (19), TKA (37), TSA (6)	>24 (24 to 84)	50
De Meo	2023	III	7	4 M, 3 W	66.9 (SD 7.7)	THA (3), TKA (4)	7.7 (SD 1.8)	54
Dimofte	2024	III	45	30 M, 15 W	66.7 (SD 11.9)	THA (45)	NA	46
Sigmund	2024	III	102	42 M, 60 W	76 (67 to 83)	THA (36), TKA (66)	39 (23-57)	63
**Summary**			376 (377)	226 M (60%), 151 W (40%)	70.5∗∗ (32 to 94)	THA (182, 48%), TKA (189, 50%), TSA (6, 2%)	24.8∗∗ (1.5 to 84)	31 to 63
Summary data excludes studies with no reported data. ∗Inconsistent report, ∗∗weighted mean (excluding studies with no reported data)								

**PJI prophylaxis**								
Mohamed	2022	III	48	28 M, 20 W	62.2 (SD 12.6)	THA (48)	>24	63
Zhang	2024	III	35	9 M, 26 W	64.0 (SD 7.9)	TKA (35)	3	50
**Summary**			83	37 M (45%), 46 W (55%)	63.0∗∗	THA (48, 58%), TKA (35, 42%)	3∗∗ (3 to 24)	50 to 63
Summary data excludes studies with no reported data. ∗Inconsistent report, ∗∗weighted mean (excluding studies with no reported data)								

**Mixed indications**								
McPherson	2013	IV	250	NA	NA	THA (108), TKA (142)	>3	38
Menon	2018	IV	39	28 M, 11 W	51 (10 to 79)	NA	25.7 (6 to 49)	38
Kallala	2018	IV	755	374 M, 381 W	63 (30 to 94)	THA (299), TKA (456)	35 (0 to 78)	50
Lum	2018	IV	56	NA	NA	THA (30), TKA (26)	NA	31
Vallon	2022	IV	215	138 M, 77 W	69 (5 to 90)	NA	>12	56
**Summary**			1315	540 M (54%) 469 W (46%)	63.8∗∗ (10 to 94)	THA (437, 41%), TKA (624, 59%)	34.5∗∗ (0 to 78)	31 to 56

Summary data excludes studies with no reported data.

∗Inconsistent report, ∗∗weighted mean (excluding studies with no reported data).

Abbreviations: M (men), MINORS (Methodological Index for Non-Randomized studies), n (number of cases), PJI (periprosthetic joint infection), THA (total hip arthroplasty), TKA (total knee arthroplasty), TSA (total shoulder arthroplasty), W (women).

**Table 3:** Patient infection profile and calcium sulfate treatment data.

First Author	Year	Etiology (n)	Cierny-Mader classification (n)	Isolated bacteria (n)	Calcium sulfate			Mean duration of Antibiotic Treatment, weeks (range)	Additional procedures (n)
					Product	Antibiotics (n)	Placement		
**Osteomyelitis treatment**									
McKee	2002	Posttraumatic (25)	I (1) III (6) IV (18) A (4) B (21)	*S. aureus* (9), *S. epidermidis* (4), *P. aeruginosa* (2) Polymicrobial (10)	OsteoSet-T	Tobramycin (25)	Defect	7.2 (6 to 12)	Fixation (15), Soft tissue reconstruction (6)
Gitelis	2002	NA	NA	*S. aureus* (5), Polymicrobial (1)	OsteoSet-BVF	Tobramycin (5), Tobramycin + Vancomycin (1)	Intracavitary	6	-
Chang	2007	NA	NA	NA	OsteoSet	Tobramycin (19), Vancomycin (6)	Intracavitary	≥2	-
McKee	2010	Posttraumatic (14), Other (1)	I (5) III (4) IV (6) A (3) B (12)	*S. aureus* (3), *S. epidermidis* (3), Other (3) Polymicrobial (6)	OsteoSet-T	Tobramycin (15)	Intracavitary	4.6	-
Humm	2014	Posttraumatic (21)	III (7) IV (14) A (19) B (2)	*S. aureus* (4), CoNS (4), Other (6), Polymicrobial (4), Culture negative (3)	OsteoSet-T	Tobramycin (21)	Defect/intramedullary	(6 to 26.1)	Fixation (9), Soft tissue reconstruction (7)
Ferguson	2014	Hematogenous (57), Posttraumatic (110), Postoperative (23), Other (5)	I (12) II (1) III (144) IV (38) A (69) B (125) C (1)	*S. aureus* (49), MRSA (7), CoNS (10), *P. aeruginosa* (9), *K. pneumoniae* (1), *E. coli* (4), *E. cloacae* (3), Other (13) Polymicrobial (31), Culture negative (68)	OsteoSet-T	Tobramycin (195)	Defect	≥6 (most patients)	Fixation (49), Soft tissue reconstruction (46)
Ferrando	2017	Posttraumatic (2), Postoperative (11)	NA	*S. aureus* (5), MRSA (2), *P. aeruginosa* (1), *E. coli* (1), *E. cloacae* (1), Other (2), Polymicrobial (1)	Stimulan	Vancomycin + Gentamicin (13)	Intracavitary	8.9 (SD 1.7)	Soft tissue reconstruction (1)
Masrouha	2018	Posttraumatic (13)	IV (13)	*S. aureus* (2), CoNS (5), *E. coli* (2), *E. cloacae* (1), Polymicrobial (3)	Stimulan	Vancomycin + Gentamicin (13)	Defect	(0.4 to 0.7)	Fixation (13)
Badie	2019	Hematogenous (17), Posttraumatic (13)	NA	*S. aureus* (15), MRSA (3), *K. pneumoniae* (2), *E. coli* (2), Other (4), Polymicrobial (2), Culture negative (2)	Stimulan	Vancomycin + Gentamicin (30)	Defect	6	NA
Qin	2020	Posttraumatic (30), Other (3)	NA	*S. aureus* (7), *P. aeruginosa* (5), *E. coli* (1) *E. faecalis* (1), *E. cloacae* (2), Other (4), Polymicrobial (3), Culture negative (10)	Stimulan	NA	Defect	≤2	Soft tissue reconstruction (3)
Zhou	2020	Hematogenous (10), Posttraumatic (31), Other (2)	III (43), A (36), B (7)	*S. aureus* (11), *P. aeruginosa* (2), *K. pneumoniae* (1), *E. coli* (1), *E. faecalis* (1), *E. cloacae* (1), Other (3), Polymicrobial (1), Culture negative (22)	Stimulan	Vancomycin + Gentamicin (43)	Defect	≤2	Fixation (13), Soft tissue reconstruction (2)
Jiang	2020	Hematogenous (4), Posttraumatic (30)	III (34) A (3) B (31)	*S. aureus* (2), *P. aeruginosa* (5), *E. coli* (1), *E. cloacae* (2), Other (2), Polymicrobial (2), Culture negative (20)	Stimulan	Vancomycin + Gentamicin (34)	Defect	6	Soft tissue reconstruction (3)
Zhao	2020	Hematogenous (4), Posttraumatic (6)	NA	*S. aureus* (5), *E. cloacae* (1), Culture negative (4)	OsteoSet	Vancomycin (10)	Defect	6.5 (SD 1.3)	Fixation (2), Soft tissue reconstruction (0)
Ruan	2021	Posttraumatic (35)	III (35) A (20) B (15)	*S. aureus* (4), *S. epidermidis* (1), *P. aeruginosa* (1), *K. pneumoniae* (2), *E. coli* (1), *E. faecalis* (1), Other (3), Polymicrobial (1), Culture negative (21)	OsteoSet	Vancomycin + Gentamicin (35)	Defect	6	Fixation (4, change from internal to external fixation), Soft tissue reconstruction (35)
Xu	2022	Posttraumatic (35), Other (6)	NA	*S. aureus* (9), *P. aeruginosa* (5), *K. pneumoniae* (1), *E. coli* (1), *E. faecalis* (2), *E. cloacae* (2), Other (8), Culture negative (13)	NA	NA	Defect	NA	NA
Patel	2023	Posttraumatic (13)	NA	*S. aureus* (5), MRSA (1), *S. epidermidis* (1), *E. coli* (1), Other (2), Culture negative (3)	Stimulan	Vancomycin + Gentamicin (13)	Intramedullary	6.3 (2 to 12)	Fixation (3), Soft tissue reconstruction (2)
McKee	2023	NA	NA	NA	OsteoSet	NA	NA	NA	NA
Du	2023	NA	NA	NA	NA	Vancomycin (44), Gentamicin (39) Other (62)	Defect	NA	Soft tissue reconstruction (35)
Casiraghi	2023	Posttraumatic (5), Postoperative (2)	NA	*S. aureus* (2), *K. pneumoniae* (1), *E. faecalis* (1), Other (1), Culture negative (2)	Stimulan	Vancomycin + Gentamicin (7)	Near implant	12	NA
Mereddy	2023	Hematogenous (28), Postoperative (78)	I (8) II (4) III (82) IV (6) A (40) B (44) C (16)	*S. aureus* (28), MRSA (2), *S. epidermidis* (1), *P. aeruginosa* (5), *K. pneumoniae* (13), *E. coli* (5), *E. faecalis* (5), Other (7), Polymicrobial (8), Culture negative (27)∗	Stimulan	NA	NA	NA	Soft tissue reconstruction (1)
Selvaratnam	2023	Hematogenous (6), Postoperative (28)	I (21) III (13) A (30) B (4)	*S. aureus* (18), MRSA (1), CoNS (6), *P. aeruginosa* (1), Other (4), Polymicrobial (10), Culture negative (4)∗	Stimulan	Gentamicin (34)	NA	6	Soft tissue reconstruction (2)
Palo	2024	NA	NA	*S. aureus* (46), MRSA (28), *S. epidermidis* (9), *P. aeruginosa* (8), *K. pneumoniae* (4)	Stimulan	Vancomycin + Gentamicin (32), Other (63)	Defect	(4 to 6)	NA
Su	2024	Posttraumatic (91), Postoperative (32), Other (40)	III (163)	*S. aureus* (82), MRSA (22), *S. epidermidis* (11), *P. aeruginosa* (34), *E. coli* (8), Culture negative (6)	NA	NA	Defect	NA	Soft tissue reconstruction (45)
**Summary**		Hematogenous (98, 12%), Posttraumatic (474, 57%), Postoperative (174, 21%), Other (85, 10%)	I (47, 7%), II (5, 1%), III (531, 78%), IV (95, 14%), A (224, 45%), B (261, 52%), C (17, 3%)	*S. aureus* (311, 33%), MRSA (66, 7%), CoNS (25, 3%), *S. epidermidis* (30, 3%), *P. aeruginosa* (78, 8%), *K. pneumoniae* (25, 3%), *E. coli* (28, 3%), *E. faecalis* (11, 1%), *E. cloacae* (13, 1%), Other (62, 7%), Polymicrobial (83, 9%), Culture negative (205, 22%)∗	Osteoset (9 studies, 45%; 438 procedures, 51%), Stimulan (11 studies, 55%; 421 procedures, 49%)	Tobramycin (280, 37%), Vancomycin (60, 8%), Gentamicin (73, 10%), Vancomycin + Gentamicin (220, 29%), Other (126, 17%)		6.4∗∗ (0.4 to 26.1)	Fixation (108, 28%), Soft tissue reconstruction (188, 21%)
Summary data excludes studies with no reported data. ∗Inconsistent report, ∗∗weighted mean (excluding studies with no reported data)									

**PJI treatment**									
Kallala	2015	NA	NA	*S. aureus* (3), *S. epidermidis* (4), P aeruginosa (1), *E. faecalis* (1), Other (3), Polymicrobial (3)	Stimulan	Vancomycin + Gentamicin (15)	Around joint	6	Hip resurfacing to THA (2), THA revision (7), TKA revision (6)
Flierl	2017	Hematogenous (19), Postoperative (14)	NA	*S. aureus* (13), MRSA (3), *S. epidermidis* (4), *E. coli* (1), Other (8), Polymicrobial (1), Culture negative (3)	Stimulan	Tobramycin + Vancomycin (33)	Deep wound	≥6	DAIR (33)
Sandiford	2020	NA	NA	NA	Stimulan	NA	Intracapsular	≥6	1-stage (7), First-stage (4), Second stage (9), DAIR (6), Excision arthroplasty (1), Periprosthetic fracture (2)
Tarity	2022	NA	NA	*S. aureus* (7), MRSA (4), *S. epidermidis* (2), *E. coli* (1), *E. cloacae* (1), Other (3), Culture negative (2)	NA	Tobramycin + Vancomycin (18), NA (2)	Intracapsular	≥6	DAIR (20)
Piovan	2022	Hematogenous (5), Postoperative (12)	NA	*S. aureus* (1), CoNS (6), *K. pneumoniae* (1), *E. coli* (1), C. acnes (1), Other (4), Polymicrobial (1), Culture negative (2)	Stimulan	Tobramycin + Vancomycin (4), Vancomycin + Gentamicin (13)	Intracapsular	11.6 (8 to 14)	DAIR (17)
Reinisch	2022	NA	NA	*S. aureus* (5), CoNS (10), *P. aeruginosa* (2), *E. coli* (2), C. acnes (1), Other (11) Polymicrobial (2), Culture negative (2)∗	Osteoset	Tobramycin (2), Vancomycin (23), Other (2)	Intracapsular	12	DAIR (27)
Iorio	2023	NA	NA	*S. aureus* (7), MRSA (6), CoNS (4), Other (3)	Stimulan	NA	NA	7.1 (SD 1.8)	First stage of 2-stage revision with implantation of antibiotic-loaded cement spacer (20)
Indelli	2023	NA	NA	NA	Stimulan	NA	Intracapsular	≥12	DAIR (62)
De Meo	2023	Hematogenous (4), Postoperative (3)	NA	*S. aureus* (2), CoNS (1), *P. aeruginosa* (1), *E. coli* (1), Polymicrobial (1), Culture negative (1)	Stimulan	Vancomycin + Gentamicin (6), Other (1)	Intracapsular	12	DAIR (7)
Dimofte	2024	NA	NA	NA	Stimulan	Vancomycin + Gentamicin (45)	Intracapsular	6	DAIR (26), First stage of 2-stage revision (19)
Sigmund	2024	Hematogenous (28), Postoperative (30), Chronic (44)	NA	*S. aureus* (30), CoNS (15), *P. aeruginosa* (2), Other (23), Polymicrobial (26), Culture negative (6)	Stimulan	Vancomycin + Gentamicin (99), Other (3)	Intracapsular	(12 to 24)	DAIR (102)
**Summary**		Hematogenous (56, 35%), Postoperative (59, 37%), Chronic (44, 28%)		*S. aureus* (68, 28%), MRSA (13, 5%), CoNS (36, 15%), *S. epidermidis* (10, 4%), *P. aeruginosa* (6, 2%), *K. pneumoniae* (1, 0.4%), *E. coli* (6, 2%), *E. faecalis* (1, 0.4%), *E. cloacae* (1, 0.4%), C. acnes (2, 1%), Other (55, 23%), Polymicrobial (34, 14%), Culture negative (16, 7%)	Osteoset (1 study, 10%, 27 procedures, 8%), Stimulan (9 studies, 90%; 330 procedures, 92%)	Tobramycin (2, 1%), Vancomycin (23, 9%), Tobramycin + Vancomycin (55, 21%), Vancomycin + Gentamicin (178, 67%), Other (6, 2%)		8.5∗∗ (6 to 24)	DAIR (300, 80%), Infected revision TJA (77, 20%)
Summary data excludes studies with no reported data. ∗Inconsistent report, ∗∗weighted mean (excluding studies with no reported data)									

**PJI prophylaxis**									
Mohamed	2022	PJI prophylaxis (48)	NA	NA	Stimulan	Tobramycin + Vancomycin (48)	Intracapsular	NA	1-stage aseptic revision (48)
Zhang	2024	PJI prophylaxis (35)	NA	NA	Stimulan	Vancomycin (35)	Intramedullary (femur)	0.3	Primary TKA (35)
**Summary**		PJI prophylaxis (83, 100%)			Stimulan (2 studies, 100%; 83 patients, 100%)	Tobramycin + Vancomycin (48, 58%), Vancomycin (35, 42%)		0.3	Primary TJA (35, 42%), Revision TJA (48, 58%)
Summary data excludes studies with no reported data. ∗Inconsistent report, ∗∗weighted mean (excluding studies with no reported data)									

**Mixed indications**									
McPherson	2013	PJI (126), PJI prophylaxis (124)	NA	NA	Stimulan	Tobramycin + Vancomycin (250)	Intracapsular/around joint	NA	Aseptic revision THA (58), DAIR THA (8), Resection THA (24), Reimplantation THA (18), Aseptic revision TKA (66), DAIR TKA (16), Resection TKA (35), Reimplantation TKA (25)
Menon	2018	Posttraumatic (17), Infection, other (19), PJI (3)	NA	*S. aureus* (16), CoNS (2), *P. aeruginosa* (5), *E. coli* (7), Other (3), Polymicrobial (5), Culture negative (7)∗	Stimulan	Vancomycin (17), Vancomycin + Gentamicin (4), Other (19)∗	NA	6	Soft tissue reconstruction (6)
Kallala	2018	PJI (387), PJI prophylaxis (368)	NA	NA	Stimulan	Tobramycin + Vancomycin (755)	Intracapsular	NA	Aseptic revision TJA (368), DAIR (68), First stage of 2-stage revision (176), Second stage of 2-stage revision (143)
Lum	2018	PJI (14), PJI prophylaxis (42)	NA	NA	Stimulan	Tobramycin + Vancomycin + Cefazolin (56)	Wound	NA	Primary THA (5), Aseptic revision THA (19), Infected revision THA (6), Primary TKA (6), Aseptic revision TKA (12), Infected revision TKA (8)
Vallon	2022	PJI (127), Infection, other (88)	NA	NA	Osteoset	NA	NA	NA	NA
**Summary**		Infection, posttraumatic (17, 1%), Infection, other (107, 8%), PJI (657, 50%), PJI prophylaxis (534, 41%)		*S. aureus* (16, 36%), CoNS (2, 4%), *P. aeruginosa* (5, 11%), *E. coli* (7, 16%), Other (3, 7%), Polymicrobial (5, 11%), Culture negative (7, 16%)∗	Osteoset (1 study, 20%; 215 procedures, 16%) Stimulan (4 studies, 80%; 1100 procedures, 84%)	Vancomycin (17, 2%), Tobramycin + Vancomycin (1005, 91%), Vancomycin + Gentamicin (4, 0.4%), Other (75, 7%)∗		6∗∗	Soft tissue reconstruction (6, 0.5%), Primary TJA (11, 1%) Aseptic revision TJA (523, 48%) DAIR (92, 8%) Infected revision TJA 435, 40%)
Summary data excludes studies with no reported data. ∗Inconsistent report, ∗∗weighted mean (excluding studies with no reported data)									
									

Abbreviations: CoNS (coagulase-negative staphylococcus), MRSA (methicillin-resistant *S. aureus*), n (number of cases), PJI (periprosthetic joint infection), THA (total hip arthroplasty), TKA (total knee arthroplasty), DAIR (Debridement, Antibiotics and Implant Retention), TJA (total joint arthroplasty).

#### 3.2.2 PJI treatment

The analysis included 11 studies with a total of 376 patients (377 arthroplasties) and a weighted mean follow-up of 24.8 months (range, 1.5 to 84). Affected arthroplasties included TKA in 50% (189/377) and THA in 48% (182/377) of cases (Table 2). The most commonly isolated bacteria were *S. aureus* (68/241, 28%) and coagulase negative staphylococci (36/241, 15%). CS was implanted for DAIR procedures in 80% (300/377) and infected revisions of total joint arthroplasty (TJA) in 20% (77/377) of cases. All studies administered antibiotics postoperatively for a weighted mean of 8.5 weeks (range, 6 to 24) (Table 3).

#### 3.2.3 PJI prophylaxis

Prophylactic CS use in aseptic procedures to reduce PJI incidence was reported in only two studies with a total of 83 patients and a weighted mean follow-up of 3 months (Table 2). Cases were distributed between revisions (48/83, 58%) and primary TJA (35/83, 42%) (Table 3).

#### 3.2.4 Mixed indications

Five studies encompassed 1315 patients with a weighted mean follow-up duration of 34.5 months (range, 0 to 78) (Table 2). Patients received CS for PJI treatment in 50% (657/1315) of cases, while 41% (534/1315) had CS for PJI prophylaxis (Table 3).

### 3.3 Outcomes

#### 3.3.1 Osteomyelitis treatment

**Fig. 2:**
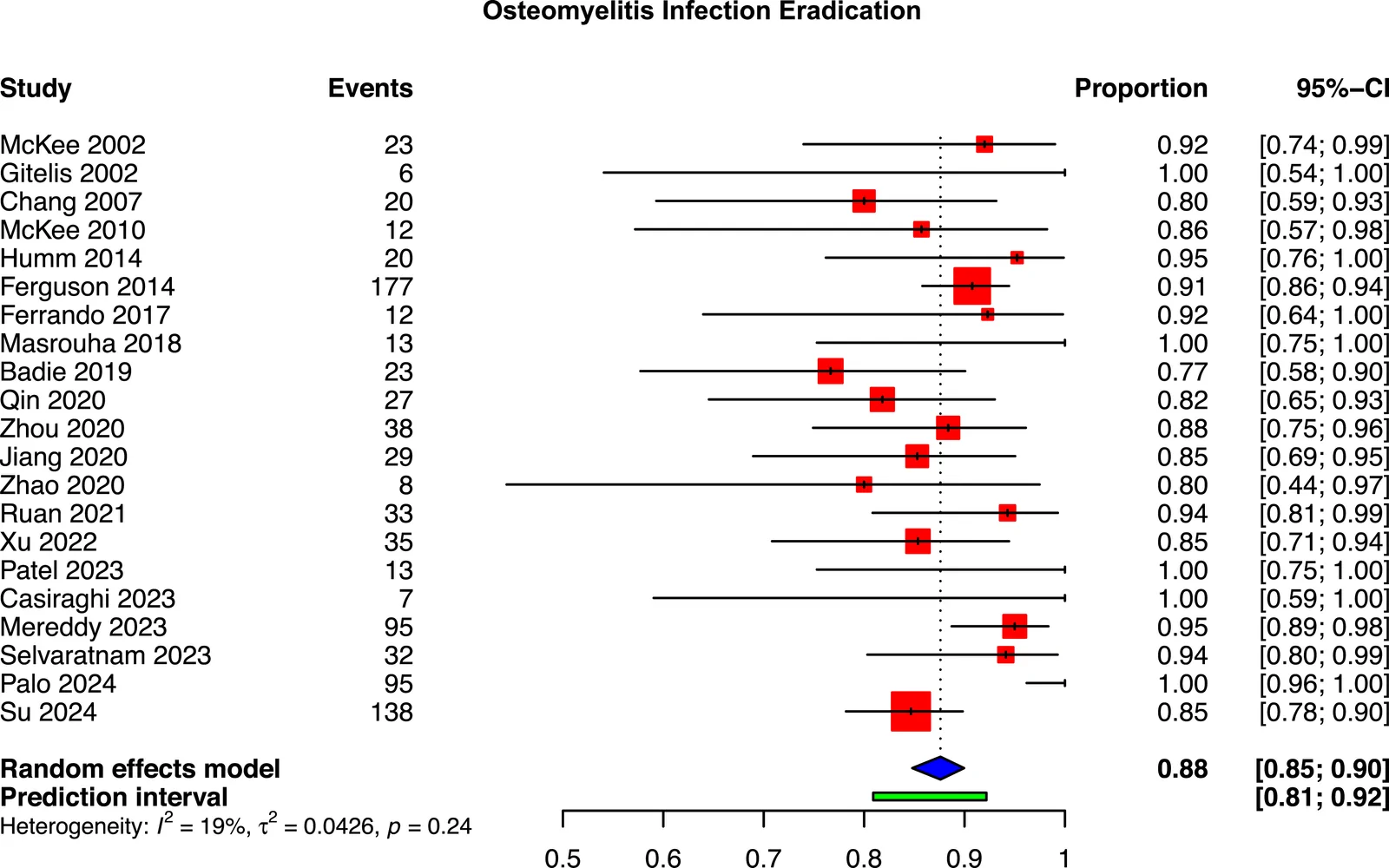
Osteomyelitis infection eradication rate. The forest plot shows infection eradication rates for osteomyelitis. The size of squares represents the weight of each study and I^2^ represents heterogeneity.

**Fig. 3:**
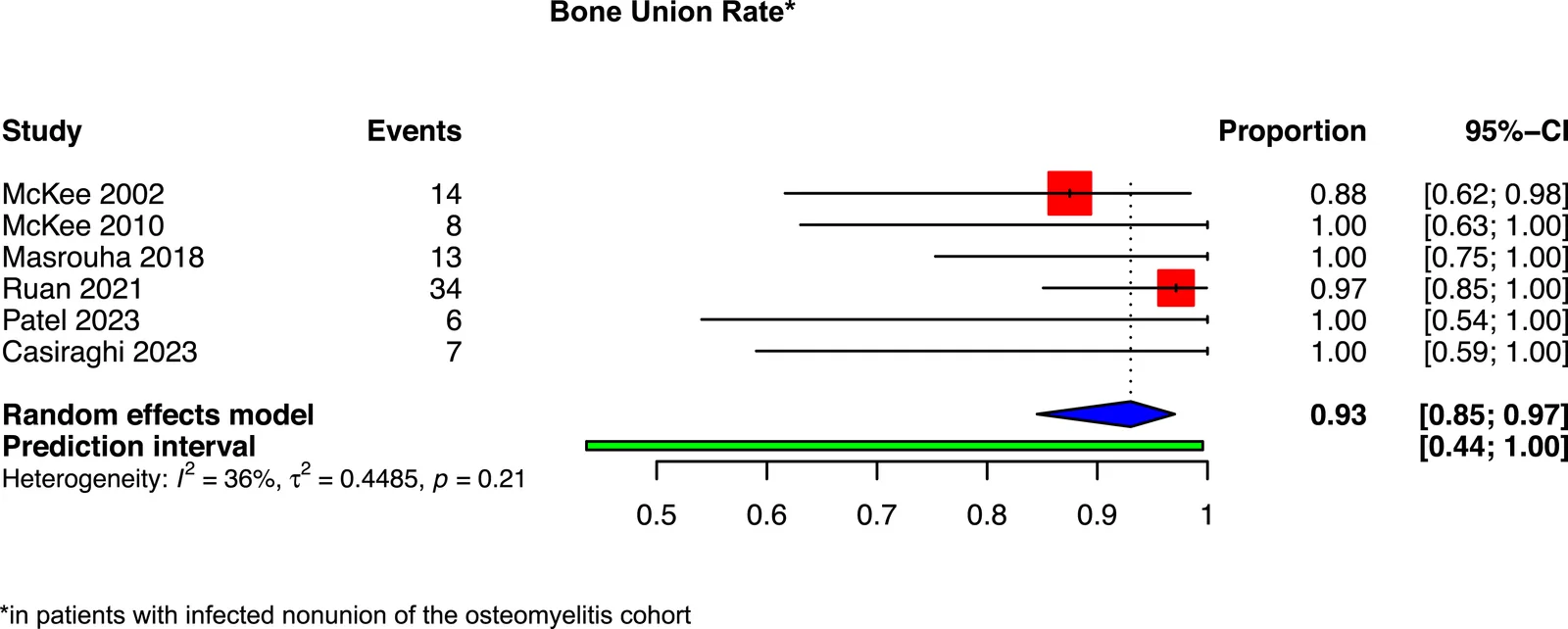
Bone union rate for infected nonunions. The forest plot shows bone union rate for infected nonunions in the osteomyelitis cohort. The size of squares represents the weight of each study and I^2^ represents heterogeneity.

The pooled infection eradication rate for osteomyelitis was 88% (95% CI: 85 to 90%; 21 studies, n = 950) with low data heterogeneity (I^2^ = 19%, *P* = 0.24) (Fig. 2). Pooled bone union rate for infected nonunions was 93% (95% CI: 85 to 97%; 6 studies, n = 85; I^2^ = 36%, *P* = 0.21) (Fig. 3). Complete CS resorption occurred in all cases after a weighted mean of 2.4 months. Wound-related complications occurred in 17% (189/1086) of cases. Reintervention was required in 13% (120/931) of patients, primarily for infection recurrence (65/87, 75%) (Table 4).

**Table 4:** Outcome data.

Osteomyelitis treatment									
First Author	Year	Infection eradication, n/N (%)	Bone union, n/N (%)	Time to bone union (months)	CS beads resorption, n/N (%)	Time to CS beads resorption (months)	Complications		Reintervention, n/N (%)
							Wound discharge and related complication, n/N (%)	Other (n)	
McKee	2002	23/25 (92)	14/16 (88)	6.9	25/25 (100)	2.7	8/25 (32)	Fracture (3)	4/25 (16)
Gitelis	2002	6/6 (100)	NA	NA	6/6 (100)	NA	0/6 (0)	Fracture (0)	0/6 (0)
Chang	2007	20/25 (80)	NA	NA	25/25 (100)	NA	NA	Fracture (0), Death (0)	3/25 (12)
McKee	2010	12/14 (86)	8/8 (100)	9	14/14 (100)	2	3/14 (21)	Fracture (2), Death (1, unrelated) Wound infection (1), Knee stiffness (1), Tibial nerve neuropraxia (1)	5/14 (36)
Humm	2014	20/21 (95)	NA	NA	NA	NA	7/21 (33)	AKI (1)	2/21 (10)
Ferguson	2014	177/195 (91)	NA	NA	NA	NA	36/195 (18)	Fracture (9), Fluid collection (9), Death (7, unrelated)	21/195 (11)
Ferrando	2017	12/13 (92)	NA	NA	NA	NA	NA	Hematoma/Seroma (2)	1/13 (8)
Masrouha	2018	13/13 (100)	13/13 (100)	5.5	NA	NA	NA	NA	NA
Badie	2019	23/30 (77)	NA	NA	30/30 (100)	2.4	NA	Fracture (1)	NA
Qin	2020	27/33 (82)	NA	NA	NA	NA	13/33 (39)	Death (1, unrelated), Fracture (0)	5/33 (15)
Zhou	2020	38/43 (88)	NA	NA	NA	NA	13/43 (30)	Pain (4), Weakness (4), Scarring (2) Joint stiffness (1), Claudication (1), Fracture (0)	8/43 (19)
Jiang	2020	29/34 (85)	NA	NA	NA	NA	11/34 (32)	Death (1, unrelated), Fracture (0)	6/34 (18)
Zhao	2020	8/10 (80)	NA	NA	10/10 (100)	NA	3/10 (30)	NA	2/10 (20)
Ruan	2021	33/35 (94)	34/35 (97)	6.2	NA	NA	1/35 (3)	Iliac anterolateral numbness (5), Iliac hematocele (2)	3/35 (9)
Xu	2022	35/41 (85)	NA	NA	NA	NA	11/41 (27)	Pain (29), Transient fever (7) Lameness (1), Fracture (0)	NA
Patel	2023	13/13 (100)	6/6 (100)	8	NA	NA	0/13 (0)	Deformity (1)	1/13 (8)
McKee	2023	NA	NA	NA	NA	NA	31/106 (29)	NA	33/106 (31)
Du	2023	NA	NA	NA	NA	NA	27/145 (19)	NA	NA
Casiraghi	2023	7/7 (100)	7/7 (100)	4.3	NA	NA	2/7 (29)	Hypercalcemia (0), HO (0)	NA
Mereddy	2023	95/100 (95)	NA	NA	NA	NA	8/100 (8)	Nonunion (4), Death (6, sepsis), Fracture (0)	4/100 (4)
Selvaratnam	2023	32/34 (94)	NA	NA	NA	NA	NA	NA	NA
Palo	2024	95/95 (100)	NA	NA	95/95 (100)	2.4	2/95 (2)	NA	2/95 (2)
Su	2024	138/163 (85)	NA	NA	NA	NA	13/163 (8)	NA	20/163 (12)
**Summary**		88% (95% CI: 85 to 90%)	93% (95% CI: 85 to 97%)	6.5∗∗	205/205 (100)	2.4∗∗	189/1086 (17)	Fracture (15/546, 3%)	120/931 (13)
Summary data excludes studies with no reported data. ∗Inconsistent report, ∗∗weighted mean (excluding studies with no reported data)									

**PJI treatment**									
Kallala	2015	14/15 (93)	NA	NA	15/15 (100)	1	0/15 (0)	Hypercalcemia (3, 1 symptomatic)	NA
Flierl	2017	17/33 (52)	NA	NA	NA	NA	NA	NA	7/33 (21)
Sandiford	2020	NA	NA	NA	29/29 (100)	1.5	1/29 (3)	Hypercalcemia (0), HO (0)	1/29 (3)
Tarity	2022	11/20 (55)	NA	NA	NA	NA	NA	NA	NA
Piovan	2022	15/17 (88)	NA	NA	NA	NA	0/17 (0)	Hypercalcemia (0), HO (0)	NA
Reinisch	2022	23/27 (85)	NA	NA	NA	NA	NA	NA	4/27 (15)
Iorio	2023	20/20 (100)	NA	NA	NA	NA	NA	NA	0/20 (0)
Indelli	2023	48/62 (77)	NA	NA	NA	NA	4/62 (6)	HO (1), Hypercalcemia (0)	14/62 (23)
De Meo	2023	7/7 (100)	NA	NA	NA	NA	0/7 (0)	Hypercalcemia (0), HO (0)	0/7 (0)
Dimofte	2024	42/45 (93)	NA	NA	NA	NA	1/45 (2)	Hypercalcemia (0), HO (0)	NA
Sigmund	2024	65/102 (64)	NA	NA	NA	NA	17/102 (17)	Hypercalcemia (9, 1 symptomatic), HO (8), Death (19, 3 due to PJI)	35/102 (34)
**Summary**		88% (95% CI: 76 to 95%) DAIR: 269/360 (75); Two-stage revision, first stage: 191/196 (97); Two-stage revision, second stage: 168/186 (90); Resection arthroplasty: 57/59 (97)			44/44 (100)	1.3∗∗	23/277 (8)	Hypercalcemia (12/277, 4%), HO (9/262, 3%)	61/280 (22)
Summary data excludes studies with no reported data. Meta-analysis result for infection eradication includes above studies plus PJI eradication data from studies with mixed indications (McPherson 2013, Kallala 2018, Lum 2018; see Table 4, “Mixed indications” section). ∗Inconsistent report, ∗∗weighted mean (excluding studies with no reported data)									

**PJI prophylaxis**									
Mohamed	2022	3/48 (6) (incidence)	NA	NA	NA	NA	NA	NA	NA
Zhang	2024	0/35 (0) (incidence)	NA	NA	NA	NA	NA	NA	NA
**Summary**		3/83 (4) 16/617 (3)◊							
Summary data excludes studies with no reported data. ∗Inconsistent report, ∗∗weighted mean (excluding studies with no reported data), ◊descriptive summary includes above studies plus PJI incidence data from studies with mixed indications (McPherson 2013, Kallala 2018, Lum 2018; see Table 4, “Mixed indications” section).									

**Mixed indications**									
McPherson	2013	119/126 (94, eradication) ‡, 6/124 (5, incidence) ◊	NA	NA	NA	NA	8/250 (3)	AKI (2), HO (3), Fracture (1), Death (4, unspecified)	20/250 (8)
Menon	2018	34/39 (87, eradication)	7/8 (88)	NA	39/39 (100)	1.2	8/39 (21)	Death (2, sepsis)	7/39 (18)
Kallala	2018	360/387 (93, eradication) ‡, 7/368 (2, incidence) ◊	NA	NA	NA	NA	32/755 (4)	Hypercalcemia (41, 2 symptomatic), HO (13), Death (14, unspecified)	59/755 (8)
Lum	2018	14/14 (100, eradication) ‡ 0/42 (0, incidence) ◊	NA	NA	56/56 (100)	NA	1/56 (2)	HO (1)	1/56 (2)
Vallon	2022	NA	NA	NA	NA	NA	NA	Hypercalcemia (2)	NA
**Summary**			7/8 (88)		95/95 (100)	1.2∗∗	49/1100 (4)	Hypercalcemia (4/970, 0.4%) HO (17/1061, 2%)	87/1100 (8)

Summary data excludes studies with no reported data.

∗Inconsistent report, ∗∗weighted mean (excluding studies with no reported data), ‡included in meta-analysis for PJI eradication (see Table 4, “PJI treatment” section), ◊included in descriptive summary for PJI incidence (see Table 4, “PJI prophylaxis” section).

Abbreviations: AKI (acute kidney injury), CS (calcium sulfate), HO (heterotopic ossification), n (number of cases), N (total sample size), PJI (periprosthetic joint infection).

#### 3.3.2 PJI treatment

**Fig. 4:**
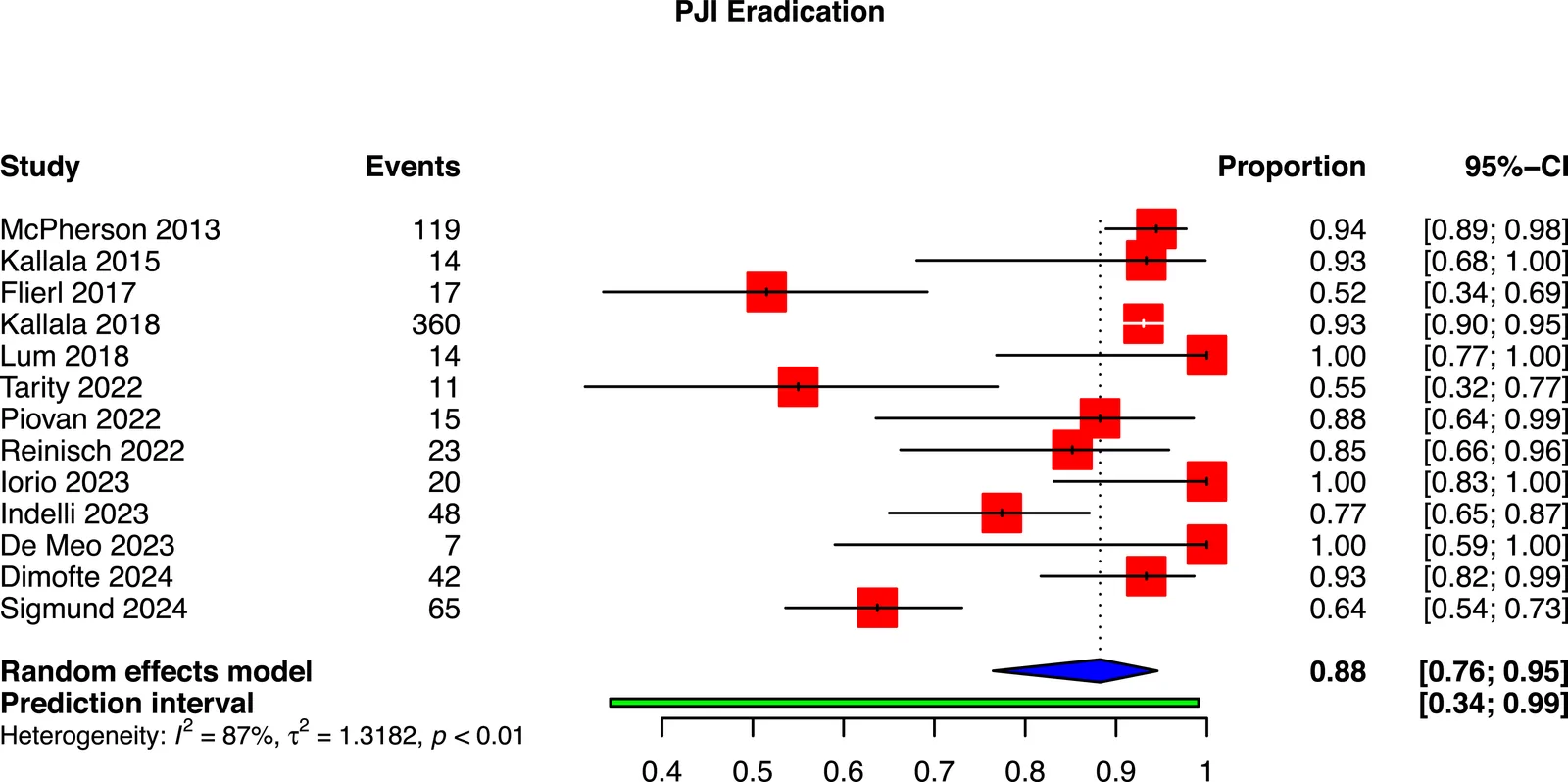
Prosthetic joint infection eradication rate. The forest plot shows PJI eradication rate. The size of squares represents the weight of each study and I^2^ represents heterogeneity.

The pooled PJI eradication rate was 88% (95% CI: 76 to 95 %; 13 studies, n = 875) with substantial data heterogeneity (I^2^ = 87%, *P* < 0.01) (Fig. 4). When analyzed by procedure type, eradication rates varied considerably: DAIR procedure achieved a 75% (269/360) success rate, two-stage revisions showed success rates of 97% (191/196) and 90% (168/186) for first and second stages respectively, while resection arthroplasty had a 97% (57/59) eradication rate. Complete CS resorption was observed in all cases after a weighted mean of 1.3 months. Wound discharge was documented in 8% (23/277) of cases. Additional complications included HO in nine cases (9/262, 3%) and hypercalcemia in 12 cases (12/277, 4%). Symptomatic hypercalcemia requiring treatment occurred in two cases. The reintervention rate was 22% (61/280), with infection recurrence accounting for 95% (21/22) of cases in which the cause for reoperation was documented (Table 4).

#### 3.3.3 PJI prophylaxis and mixed indications

Incidence of PJI when CS was used for infection prophylaxis was reported in five studies.^15^^,^^36^^,^^44^^,^^54^^,^^68^ The overall infection incidence rate was 3% (16/617). Resorption of CS was achieved in all cases. Wound-related complications were observed in 4% (49/1100) of cases, while HO occurred in 17 cases (17/1,061, 2%). Symptomatic hypercalcemia requiring treatment consisting of a combination of hydration, intravenous bisphosphonates and subcutaneous calcitonin was reported in four cases. Reintervention was necessary in 8% (87/1100) of patients. The most common indication was infection recurrence, occurring in 44% (38/87) of reinterventions. New infections in prophylaxis cases accounted for 15% of reinterventions (13/87), while 16% (14/87) were caused by prosthetic instability (Table 4).

## 4 Discussion

This systematic review of 41 studies evaluated the outcomes and safety of antibiotic-loaded CS beads as an adjunct in orthopaedic surgery. Treatment protocols incorporating CS were associated with a pooled infection eradication rate of 88% (95% CI: 85 to 90%) in osteomyelitis across 21 studies, while the pooled bone union rate reached 93% (95% CI: 85 to 97%) among 85 patients with infected nonunion in six studies. For PJI treatment, the pooled infection eradication rate was 88 % (95% CI: 76 to 95%) across 13 studies, though accompanied by considerable data heterogeneity (I^2^ = 87%, *P* < 0.01), driven in part by two studies reporting markedly low infection eradication rates.^40^^,^^52^

These two studies encompassed 53 patients who received CS combined predominantly with tobramycin and vancomycin during DAIR procedures for acute early PJI, followed by a minimum of six weeks of intravenous antibiotics. The underlying reason for this discrepancy with the literature is not entirely clear. Both Flierl et al.^40^ and Tarity et al.^52^ lacked specification of symptoms duration and procedure timing, factors affecting biofilm formation. Additionally, Tarity et al.^52^ omitted modular component exchange in 40% of cases, potentially resulting in inadequate debridement. The high *S. aureus* isolation rates in both studies (48 to 55%)^40^^,^^52^ may partially explain their lower success rates, as *S. aureus* is associated with increased DAIR failure.^72^ Similarly, Sigmund et al.^71^ reported an infection eradication rate of 64% (65/102) in the largest DAIR cohort with CS beads to date, with a median follow-up of 39 months. Notably, 43% of cases were chronic PJI, which carried a substantially higher reinfection rate compared with early postoperative infections. The longer follow-up and high proportion of chronic cases likely contributed to the lower observed eradication rate in this cohort.

The pooled bone union rate of 93% (95% CI: 85 to 97%) for infected nonunions is consistent with rates reported for conventional approaches,^73^^,^^74^ though the osteoconductive properties of CS may provide additional benefit as a scaffold for bone regeneration.

Evidence for PJI prophylaxis remains limited. Five studies reported an overall infection rate of 3% (16/617) when CS was used for prophylactic purposes, primarily in aseptic revision arthroplasties (571/617, 93%). However, these results must be interpreted with caution. First, distinguishing between persisting occult infections and new infections in revision arthroplasty remains challenging, particularly given that negative preoperative or perioperative aspiration was documented only in 172 of 571 (30%) aseptic revision procedures. Second, the lack of high-quality data precluded meaningful statistical pooling of prophylaxis outcomes. Furthermore, both studies analyzing exclusively aseptic procedures^54^^,^^68^ found no statistically significant differences in infection incidence between CS and controls.

Overall, the interpretation of these pooled estimates requires considerable caution. The outcomes reported above reflect the combined effect of multimodal treatment protocols – including patient selection, thoroughness of surgical debridement, and systemic antibiotic regimens – rather than the isolated contribution of CS beads. In the absence of controlled comparisons, it remains undetermined whether the observed eradication and union rates would have differed without local CS application. The pooled proportions presented should therefore be understood as descriptive summaries of outcomes within CS-augmented protocols, not as evidence of independent therapeutic efficacy.

Despite these interpretative constraints, certain material properties of CS can be assessed independently of comparative efficacy data. Complete CS resorption was consistently achieved, representing a major advantage over non-absorbable antibiotic carriers such as PMMA. This eliminates the need for secondary surgical procedures to remove foreign material and minimizes complications from permanent implants.

Wound-related complications occurred in 17% (189/1086) of osteomyelitis cases. This phenomenon constitutes one of the primary concerns regarding CS use in surgical applications.^15^ A systematic review of comparative studies^21^ found higher wound leakage rates with CS in the management of osteomyelitis compared to other methods (wound irrigation-suction, PMMA beads or spacer, bioactive glass S53P4), though the difference did not reach statistical significance. Various factors have been discussed as possibly contributing to prolonged wound discharge, including CS composition and implanted amount.^36^ Du et al.^61^ investigated risk factors for serous exudation following treatment of fracture-related infections with additional antibiotic-loaded CS beads, identifying CS amount, combined flap surgery, and thinner soft tissue coverage as statistically significant predictors of serous exudation. In contrast, PJI treatment with CS demonstrated a markedly lower wound complication rate of only 8% (23/277), with most cases coming from a single study.^71^ PJI procedures allow intracapsular CS placement beneath a substantially thicker soft tissue envelope. Conversely, osteomyelitis cases, particularly those involving subcutaneous bone such as the tibia, have inherently limited soft tissue coverage and may demonstrate increased susceptibility to wound discharge.

Despite this substantial incidence, particularly in osteomyelitis cases, the clinical relevance of exudation following CS implantation requires careful interpretation. Authors advocated conservative management through frequent sterile dressing changes, with spontaneous resolution reported in most cases.^32^^,^^48^^,^^49^^,^^55^^,^^63^ Ferguson et al.^38^ demonstrated that postoperative wound leakage was not predictive of treatment failure, further supporting a conservative approach. Therefore, wound exudation should be evaluated in conjunction with additional clinical parameters rather than interpreted as definitive evidence of treatment failure or recurrent infection.

Beyond wound-related issues, CS demonstrated a favorable safety profile. Hypercalcemia occurred in 4% of PJI cases, though the majority were asymptomatic. A systematic review by Tarar et al.^20^ reported a hypercalcemia rate of 4.2% (44/1049), with only 0.3% (3/1049) of patients requiring treatment. Due to the low incidence and limited reporting of patient-specific risk factors for hypercalcemia, the optimal screening protocol to identify high-risk patients remains undefined. However, Kallala et al.^15^ observed that patients who developed hypercalcemia received larger beads volumes compared to those without complications, suggesting a dose-dependent relationship. These findings indicate that while hypercalcemia represents a recognized complication, the incidence of symptomatic hypercalcemia requiring therapeutic intervention remains low. Similarly, isolated cases of HO were documented in the reviewed literature. Given the substantially higher rates of HO reported in THA literature,^75^ there is no evidence to suggest that CS contributes to HO formation.

Several systematic reviews have previously examined antibiotic-loaded CS in orthopaedic infections, though each with a narrower scope. Abosala and Ali^19^ focused exclusively on PJI, synthesizing five studies, while Sheridan et al.^21^ conducted a meta-analysis limited to comparative studies on chronic osteomyelitis, also including five studies. Other reviews similarly concentrated on either osteomyelitis or infected nonunion and fracture-related infections.^22^^,^^24^ Notably, none of these reviews evaluated the use of CS for infection prophylaxis in aseptic procedures, which represents a growing area of clinical interest given the increasing volume of revision arthroplasty. The present review consolidates outcomes across the full spectrum of CS indications – osteomyelitis, infected nonunion, PJI treatment, and prophylaxis – within a single analysis of 41 studies, providing the most comprehensive overview of both efficacy and safety to date.

The following limitations should be considered when interpreting the current findings. Most studies were observational and of retrospective design with small constituent sample size despite the rather large overall sample size. Critically, no randomized controlled trials comparing the use of CS beads with alternative treatments and only two level II studies were identified, precluding causal inference regarding efficacy. Methodological inconsistencies and reporting deficiencies were frequent, as shown by the MINORS score results, and should be considered when interpreting the pooled estimates. Publication bias was not formally assessed through funnel plot analysis or statistical tests for small-study effects, which may have influenced the pooled estimates. The substantial variation in follow-up duration may have influenced reported infection eradication rates, as longer follow-up periods allow detection of late recurrence events that would be missed in shorter-term studies. Primary outcome definitions, particularly for infection eradication, varied across studies. For example, some studies defined eradication as absence of clinical signs of infection at final follow-up, while others required additional normalization of inflammatory markers. This variability necessitated reliance on individual authors’ criteria rather than standardized definitions. Substantial data heterogeneity was found in terms of clinical scenario, CS volume, surgical delivery site, choice of combined antibiotic/antifungal substance and additional systemic antibiotic therapy, which limited the feasibility of more detailed meta-analysis and precluded meaningful subgroup analysis. Additionally, no quantitative data on cost-effectiveness was reported.

## 5 Conclusion

Current evidence describes favorable infection eradication rates when antibiotic-loaded CS beads are used as an adjunct in orthopaedic infection management and prevention. However, the absence of randomized comparative trials precludes conclusions regarding independent efficacy. All studies showed complete CS resorption. The incidence of wound-related complications was substantial in osteomyelitis cases, while other adverse events were rare. Higher-quality comparative studies with standardized outcome definitions are required to better define the clinical role of CS as a biodegradable antibiotic delivery system and to determine whether routine adoption can be recommended.

## Guardian/patient's consent

Not applicable.

## CRediT authorship contribution statement

Alberto Pedrazzini: Writing — original draft, Writing — review & editing, Software, Resources, Project administration, Methodology, Investigation. Vishwa Suravaram: Writing — review & editing, Visualization, Validation, Supervision, Software, Resources, Data curation. Victor Yan Zhe Lu: Writing — review & editing, Visualization, Software, Resources, Project administration, Investigation, Formal analysis. Armando Hoch: Writing — review & editing, Validation, Supervision, Resources, Project administration, Methodology, Investigation, Data curation. Patrick O. Zingg: Writing — review & editing, Visualization, Resources, Project administration, Methodology, Investigation. Octavian Andronic: Writing — review & editing, Supervision, Software, Resources, Project administration, Methodology, Formal analysis.

## Ethics approval and consent to participate

Not applicable. This systematic review analyzed previously published data and did not involve direct patient contact or new data collection from human participants.

## Availability of data and materials

All study associated data are stored on a local storage in the institutional database and are password-protected. These data can be accessed and reused. Requests should be forwarded to the corresponding author.

## Ethics approval and consent to participate

Not applicable. This systematic review analyzed previously published data and did not involve direct patient contact or new data collection from human participants.

## Funding

This research did not receive any specific grant from funding agencies in the public, commercial, or not-for-profit sectors.

## Declarations of interest

None.

## Legend of abbreviations

AKI: Acute kidney injury
CI: Confidence interval
CS: Calcium sulfate
DAIR: Debridement, antibiotics and implant retention
HO: Heterotopic ossification
MRSA: Methicillin-resistant *Staphylococcus aureus*
PJI: Periprosthetic joint infection
PMMA: Polymethyl methacrylate
THA: Total hip arthroplasty
TJA: Total joint arthroplasty
TKA: Total knee arthroplasty
